# The Atlanta Medical Society

**Published:** 1882-03

**Authors:** 


### THE ATLANTA MEDICAL SOCIETY.

*Reported for the Atlanta Medical Register.*

ATLANTA, January 21, 1882.

The President, Dr. W. S. Armstrong, in the chair.

Dr. Wolff, United States army, reported a case of knife wound of the liver. The subject was a policeman who re ceived the stab while in the discharge of his duties. The wound penetrated the right lobe and the organ protruded slightly through the incision. It could be distinctly made out by the touch. The hemorrhage, which was considerable at first, yielded to the application of cold. Tested some of the blood and found that it contained sugar. Gave quinine, and hypodermic injections of morphine to relieve pain. Placed a tent in the wound to force granulation from the bottom. Febrile reaction not very great. Stools of light color. On the third day tympanitis supervened and the whole skin became icterous—this was intensely marked for a considerable area around the wound—the conjunctiva yellow, and there was retention of urine, which necessitated the use of the catheter. Urine dark colored and turbid. Noticed a twitching in the region of the wound, due, no doubt, to division of branches of the intercostal nerve. After a few days began to pass his urine naturally; the wound showed healthy granulations, and eventually he made a good recovery.

A large number of gun-shot wounds of the liver, with recovery, are on record, but very few recoveries after a stab wound are reported. This is due, he thinks, to the fact that the great danger lies in hemorrhage, which, of course, is much more serious from an incised wound than from a lacerated wound, such as is produced by a bullet.

Photographic representations of the wound, before and after healing, were exhibited.

Dr. Wolff also reported a case possessing a medico-legal interest. The scene of the tragedy was in Texas, near the Mexican border. A boy, sixteen years old, was found hanging in a barn. A handkerchief encircled his neck, and was tied to a beam above. His face was downward and was within six inches of a pile of corn-cobs, upon which his feet, legs and thighs rested. At the post mortem the stomach was found to contain only a small quantity of food; no ecchymosis on surface of lungs; face calm, no appearance of strangulation. A protuberance was noted on the back of his neck; but no extravasation or effusion of blood. This protuberance was caused by the subluxation of the second cervical vertebra, backwards. A man was suspected, and tried for the murder of the boy. There were no witnesses to the deed, but Dr. W. testified that the luxation was post mortem, as no effusion around or about the injury was found. He illustrated his views by breaking the necks of several dogs, before and after death. These specimens were presented to the jury, and invariably sustained the position of the witness. The defendant finally confessed that he killed the boy by a blow in the hypogastric region, he then suspended the body as found, and threw his whole weight upon the back. The evidence of Dr. Wolff forced the confession and secured the conviction of the criminal.

Dr. James B. Baird, reported a case of retained placenta; interesting, he thought, on account of the obscurity, at first, and the length of time the placenta remained in the uterus after the probable loss of the fœtus.

A lady, aged about forty-three years, had her last menstrual flow on April 20th. Her husband left home on 30th of the same month, but sexual intercourse was indulged between the cessation of the menses and his departure. She missed her sickness then for two months, but being caught, about the last of June in a shower of rain, while out walking, she hastily caught up her youngest child, about three years of age, in her arms and hurried home. Soon after arriving at her house she noticed a sudden flow, which continued, not excessively, but with varying freedom, for eight weeks. Prior to this she had suffered a good deal from nausea. It was at this stage of the case that she sought advice, hoping to obtain relief from the severe pains in her back, bowels and loins. As she was in constant attendance upon an ill child, no critical examination of her case was made. She thought her unusual condition was due to the "change of life." Ordered viburnum prunifolium and laudanum—forty-five minims of the fluid extract of the viburnum and fifteen minims of the laudanum at each dose. After a few doses had been taken the pain was entirely relieved and the discharge ceased. She remained perfectly comfortable for six weeks, and as the abdomen presented no appreciable enlargement she concluded that the suggestion of pregnancy was an error, and that she was correct in referring the symptoms to the "change of life." About this time she sustained a severe jolt in a street car as the wheels passed over a fragment of stone on the rail. She soon after experienced a faint, sick feeling and was taken home in the carriage of a friend. Immediately after her arrival a terrible hemorrhage occurred, the blood falling in large clots upon the floor before she could be put to bed. When Dr. B. arrived the hemorrhage had ceased, and, for the first time, he made a digital examination of the uterus. The organ was about six inches long, the os was perfectly natural and there was no hemorrhage. Her attendants were certain that nothing excepting the blood had escaped. Ordered the same dose before used. The next day a very slight discharge of blood was observed, which continued intermittently for one week. Fluid extract of ergot was then ordered. She took two doses of one drachm each. This was followed in a few hours by very severe pains in the hypogastrium and back, and a placenta was expelled without much hemorrhage. This happened on the 10th of October—nearly six months after the last regular menstruation. The placenta was about the size of a goose's egg; was folded upon itself and the membranes—in which a slit about one inch long was noted—extended over and across its concave surface. The convex or uterine surface appeared normal and as if it had just been peeled off the walls of the womb. No signs of a fœtus were found and no remains of an umbilical cord.

He thought the ovum was blighted or expelled unnoticed, four months before, at the time of the first hemorrhage, and that the placenta had remained partially attached and feebly nourished until separated and expelled as an effect of the ergot.

Dr. W. D. Bizzell made the following report:

The case he considered of interest, as it illustrates the obscurity of some of the symptoms, unless correctly interpreted, as they occur in some of the chronic forms of Bright's diseases of the kidney.

The patient, an old gentleman about 64 years of age, rather thin, pallid and weak looking, consulted him about two months ago for what he described as catarrh of the posterior nares. An examination found no very marked changes of mucous surfaces; the patient had been using a douche and salt water for some time. Remembering the obstinacy of the catarrhal fluxes of the aged, he did not hold out very flattering promises of cure, but prescribed a dilute solution of chloride of ammonium and carbolic acid, dropped on a sponge wrung out of warm water, to be inhaled, and in addition gave some general tonics. A few weeks afterward heard from him, that the catarrh was no better, and it was his belief that the disease had extended to the lungs.

A few days after this he was called to see the patient; found him in bed, cough pretty severe, but patient stated that each coughing fit excited a severe pain in his heart; also that his head felt queer, etc. He then felt convinced that he had not looked beyond the surface in this case, and that a real diagnosis had yet to be made. His complexion was pale and pasty, but no distinctive œdema any where; pulse hard and bounding; skin dry. Examination of the heart showed hypertrophy and some dilatation; respiration was a trifle harsh, but the air entered the lungs well. The patient, when not disturbed by cough or required to answer questions, would drop into a kind of stupor. The peculiar pulse, the cough, and other symptoms, at once directed my attention to the kidneys. On inquiry he found that he had to rise several times each night to urinate. A specimen was obtained, and, on testing it, found a pale urine, containing 20 per cent. of albumen; under the microscope there were seen pale hyaline and fatty granular uriniferous tubules. Here then he had a chronic disease of the kidney, and from the attendant symptoms, undoubtedly the hard contracted kidney, which had hitherto escaped the attention of the several medical gentlemen who had treated him at various times during the past years.

When one's attention is called to the array of symptoms there is no difficulty in making the diagnosis. The peculiar, irritating cough, only bringing up tough mucus, and no serious lesion of lung structure, the peculiar pulse, and the profuse flow of pale, limpid urine; if in addition the cough disappears when the bowels are freely evacuated, which your patient will often voluntarily inform you is the case, you may be sure that kidney degeneration is the fault. The cough in these cases seems to be excited by the excrementitious material which accumulates in the blood by reason of the crippled condition of the kidneys, hence the good effects of free purgation or sweating. That the hypertrophied heart may be a factor in its production there can be no doubt. The treatment in this case was, a good purge, as the bowels were constipated, and gr. iij of potass. brom., and gr. ij of chlor. ammo. in teaspoonful doses of infusion of digitalis, which promptly relieved all the more threatening symptoms.

Dr. F. H. O'Brien asked if cardiac hypertrophy was invariable, and if the flow of urine was always excessive in contracted kidney?

Dr. Bizzell replied in the affirmative to both questions, and remarked that the hypertrophied heart was a conservative provision of nature.

Dr. Wolff added that any cause which impeded the flow of the blood favored hypertrophy, and that the overaction of the heart may cause cough, so also may retained poisonous matter within the system, by irritating the respiratory centres.

---

FEBRUARY 4, 1882.

The President, Dr. W. S. Armstrong, presiding.

Dr. Baird reported a case of paraplegia, following an attack of diphtheria. A boy, aged fourteen, was referred to him by a physician of this city. He gave the following history:

In August, while at a summer resort, had a severe attack of diphtheria. When convalescence was fully established and after his return home, he lost power in both lower extremities, which soon became almost completely paralyzed. He was placed upon a tonic treatment with massage, bathing, etc., to the affected limbs.

His general health was soon fully restored, but with no discoverable improvement of the paralysis. When he came to Dr. B.'s office he was supported by a gentleman on either side, and he dragged his feet over the carpet. He was unable to stand erect or to take a single step without support. The legs were blue, cold and shrunken. No bladder trouble or evidences of central nervous lesions

Gave a favorable prognosis and commenced the daily application to the affected muscles of faradic electricity—using alternately the primary and secondary currents.

The responses to the electrical stimulation were satisfactory. The strychnine, which he had been taking for several weeks, was continued. Amendment was rapid.

In a fortnight he came to his office and ascended the stairs without assistance, and in three weeks he was dismissed, cured.

After six days the applications were made only three times a week, and at no single sitting were they used to a degree of producing fatigue. Sensation in the legs was only slightly impaired.

Dr. John G. Earnest inquired if paralysis in such cases did not usually occur earlier than several weeks after the subsidence of the disease?

Dr. Baird replied that paralysis of some part was a not infrequent sequel of this disease. Thinks it does not usually follow immediately. The muscles of phonation are frequently paretic, and he had, not long ago, a case in which hemi-chorea followed a severe attack of diphtheria.

Dr. W. D. Bizzell remarked that the paralysis was evidently peripheral. No central cause was apparent. Time tends to relieve this class of paralyses. Electrical stimulation applied at the proper time, is undoubtedly of great benefit. It supplies the necessary relief to the enfeebled parts. It is alleged that bacteria are the cause of this trouble. They may be packed in the brain and kidneys. Massage and hygiene favor their elimination. The effect of the treatment in this case was the removal of the results, not the removal of the original cause.

The absence of bladder and rectal complications was encouraging, and the final result fully justified the wisdom of the plan of treatment adopted.

Dr. G. G. Roy was consulted about six weeks ago by a lady, about forty-five years of age, on account of a tumor in the abdomen. It had existed for twelve or eighteen months, and had been pronounced ascites. Found procedentia uteri. The uterus having escaped beyond the vulva was hanging between the thighs. The neck was not elongated, but the organ was very heavy. The enlargement of the abdomen was not equal on both sides. He regarded it as an ovarian cyst. Dr. Bizzell saw the case in consultation.

He replaced the prolapsed womb but it promptly descended when the erect posture was assumed. A cotton tampon was then applied, which served the purpose of retaining the uterus in position, but the patient becoming impatient of confinement, the cotton tampon was substituted by a uterine supporter attached to a belt worn around the waist. This proved so painful that it was abandoned. He determined to aspirate the tumor, which he did, drawing off one gallon and a half of a straw-colored fluid. After this no difficulty was found in retaining the uterus, and local treatment has reduced its size and restored it to its normal state. The patient is now comfortable. Is in doubt as to the nature of the tumor, but he now inclines to the opinion that it is ascites. There is no evidence of kidney disease. The liver is enlarged and seems unnaturally hard.

Several years ago he encountered a case of procedentia of the gravid womb. The fundus was clear of the vulva, and he delivered a six or seven months child while the uterus lay between the thighs on the bed, by steadying the uterus with one hand while with the other he aided the escape of the child. The woman suffered afterwards from a terrible attack of peritonitis but recovered and has had several children since, without and unusual complications.

Dr. A. W. Calhoun said he had recently operated upon a case with successful termination which offered some points of interest A man aged forty-five years, from Alabama, consulted him for a tumor growing from the upper portion of the left orbital cavity. It had been growing for three or four years, and when seen protruded forward an inch and a half beyond the ridge of the orbit, and extended laterally from canthus to canthus. The tumor was flattened from pressure, and the eye ball was forced downward a half inch and inwards toward the nose. There was an extreme degree of tension of the optic nerve, but vision was fairly good.

The tumor was supposed to be fibroid. The operation was commenced by a free incision in the eyebrow—so that the cicatrix would be conncealed—the tumor was carefully dissected from the periosteum lining the orbit, and from its origin around the optic foramen. It was about the size of a guinea egg, pear-shaped, the apex resting in the apex of the orbital cavity. He at first thought removal of the ball would be necessary, but finally succeeded in getting out the tumor and leaving the ball, which has receded, and the contusion incident to necessary handling is subsiding. The man made a rapid recovery. The power of the superior rectus is not yet fully regained. The operation lasted one hour and a half. Cases of this character are somewhat rare. It is not likely to recur. The removal of the tumor leaving the ball in place constitutes the most important feature of the operation.

Dr. E. L. Connally reported a case of pneumonia involving the right lung, complicated by severe and persistent hiccough. The paroxysms were prolonged and very distressing. The patient dreaded their advent on account of the discomfort and actual suffering which they occasioned. This symptom continued for three days. Dr. Armstrong saw the patient in consultation. The hiccough could not be referred to exhaustion, but he ascribed it to disordered stomach. It was accompanied by nausea and vomiting. Used as medicine morphine, quinine, chloroform, etc. The irritability of the stomach precluded the regular trial or administration of the remedies. The most successful treatment was pressure over the epigastrium. Lying on the face with a pillow under the body pressing upon the stomach afforded more relief than anything else. The hiccough has disappeared, and the pneumonia is subsiding.

Dr. Calhoun said that this report called to mind an amusing case which he encountered a number of years ago. He had trephined the skull of a man for the purpose of elevating a depressed fragment of bone. About a week after the operation the man was seized with the most annoying and violent paroxysms of hiccough. A great many remedies were used without success. Chloroform narcosis, even, did not arrest the attacks. The patient was finally given up after a week's fruitless resistance. It was thought that death would soon close the scene.

As a last resort, however, it was determined to try a tight bandage around the body. It acted like a charm. The hiccough ceased at once. The result was heralded throughout the neighborhood. A large concourse of friends—male and female—gathered in his apartment, and pouring in their congratulations, rejoiced with him that he had been suddenly rescued from impending death. But, alas! in the very midst of their enthusiasm a series of rythmical explosions were heard. The hiccough had "turned downwards," so to speak, and with unvarying regularity intermittent detonations rent the air.

The room was quickly cleared, and at the urgent request of the sick man the compress was removed, as he preferred, so he said, "the other variety." His wish was gratified, but the spell was broken, the trouble gradually subsided, and the man made a good recovery.

